# Impacts of Biochar on Physical Properties and Erosion Potential of a Mudstone Slopeland Soil

**DOI:** 10.1155/2014/602197

**Published:** 2014-12-08

**Authors:** Zeng-Yei Hseu, Shih-Hao Jien, Wei-Hsin Chien, Ruei-Cheng Liou

**Affiliations:** ^1^Department of Environmental Science and Engineering, National Pingtung University of Science and Technology, Pingtung 91201, Taiwan; ^2^Department of Soil and Water Conservation, National Pingtung University of Science and Technology, Pingtung 91201, Taiwan

## Abstract

Food demand and soil sustainability have become urgent issues recently because of the global climate changes. This study aims to evaluate the application of a biochar produced by rice hull, on changes of physiochemical characteristics and erosion potential of a degraded slopeland soil. Rice hull biochar pyrolized at 400°C was incorporated into the soil at rates of 2.5%, 5%, and 10% (w/w) and was incubated for 168 d in this study. The results indicated that biochar application reduced the Bd by 12% to 25% and the PR by 57% to 92% after incubation, compared with the control. Besides, porosity and aggregate size increased by 16% to 22% and by 0.59 to 0.94 mm, respectively. The results presented that available water contents significantly increased in the amended soils by 18% to 89% because of the obvious increase of micropores. The water conductivity of the biochar-amended soils was only found in 10% biochar treatment, which might result from significant increase of macropores and reduction of soil strength (Bd and PR). During a simulated rainfall event, soil loss contents significantly decreased by 35% to 90% in the biochar-amended soils. In conclusion, biochar application could availably raise soil quality and physical properties for tilth increasing in the degraded mudstone soil.

## 1. Introduction

The problems of food requirement and land resource have become urgent issues in the recent several decades due to climate changes. Additionally, soil degradation, such as erosion, hardsetting, and desertification, poses a serious problem affecting the productivity of agricultural lands and also threatens the world's food security in the future with increasing global population [1–4]. Development of new land management techniques to effectively use soil resources and attend land sustainability is necessary in the future, particularly in subtropical/tropical regions. Biochar has attracted attention for remediation of degraded soils recently, which is a byproduct derived from a process in which biofuel is produced using anaerobic pyrolysis at high temperatures. Biochar has been considered a suitable organic amendment for improving the physical properties and maintaining the fertility of soil, particularly degraded soils in subtropical and tropical regions [5–8].

Numerous studies have indicated that biochar effectively improves soil structure [9, 10], soil aggregate stability [11–15], and porosity [16] because of its high specific surface area (SSA) and inner porous structure. Steiner et al. [17], Chan et al. [6], Laird et al. [18], and Yuan and Xu [19] have reported that fertilizer-use efficiency and crop production increase following biochar incorporation. Biochar is composed of recalcitrant carbon from microbial degradation and of a charged surface with organic functional groups; therefore, it can increase soil organic carbon (SOC) levels and C stocks [20–23]. Biochar application could produce agronomic benefits, particularly fertility maintenance, SOC sequestration, and crop production [5, 8, 24–26]. However, few studies have examined the benefits of biochar application on the hydraulic properties and erosion recalcitrant of soil.

Hardsetting mudstone soils are considered a degradation soil because the soils are difficult to cultivate until the soil profile is rewetted [27] and they exhibit very high erosion potential. Mullins et al. [28] explained that hardsetting soils set to a hard, structureless mass during drying. Hardsetting mudstone soils constitute the area of over 1000 km^2^ of land in Taiwan [29]. Chen [30] reported that approximately 8 to 10 cm of soil is eroded from the surfaces of mudstone landscapes yearly, corresponding to a soil loss rate of 160 Mg ha^−1^ per year in Taiwan.

Natural organic materials (e.g., green manure) and artificial polymers (e.g., polyacrylamides) have been used to ameliorate degraded soils [31, 32]. However, maintaining the long-term aggregate stability of soil by applying fresh organic residues is difficult because of the rapid degradation of these traditional amendments. In addition, polymer application is costly because polymers are expensive [31]. In Taiwan, techniques such as retaining walls and shotcretes are commonly used to protect soil from erosion in mudstone areas; however, these techniques are ineffective in situ. Hence, biochar may be a potential amendment that could protect hardsetting soil from rapid degradation in the long term, thus increasing the value of Taiwanese agronomy and engineering. Few published studies have focused on the influence of biochar on the hydraulic properties and erodibility of soil. The purposes of this study were to (1) explore the effects of biochar on the physical and hydraulic properties and erosion potential of the mudstone soil and (2) assess the relationship between the physical properties and erosion potential of the soil.

## 2. Materials and Methods

### 2.1. Soil and Biochar Collection

**Figure 1:**
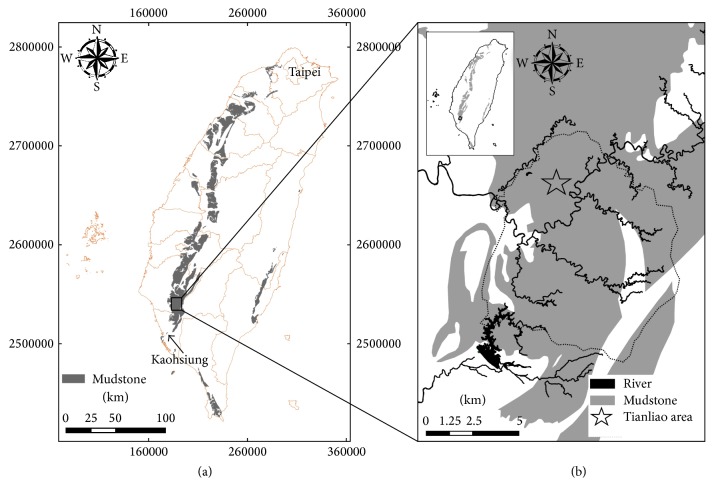
Location of soil sampling.

Mudstone occupied over than 1000 km^2^ in Taiwan [29] (Figure 1(a)). Outcrops of the mudstone formation were only found in southwestern Taiwan and occupied about 3% of land area of Taiwan. The mudstone formation formed from marine sediments from the littoral zone during the Miocene to Pliocene and Pleistocene Epochs. Sedimentary rocks created during the late Miocene Epoch to the Pleistocene Epoch can be several thousand meters deep. The geological stratigraphy is rather monotonic, consisting mainly of massive mudstones or alternating between mudstone and sandstone. In southern Taiwan, most of the mudstone slopeland soils with slope gradient below 20° are used for crop production. However, poor soil qualities, such as hardsetting, crusting, and erosion, often occur in these mudstone slopelands and result in poor production. Moreover, mudstone landscapes are characterized by sparse vegetation and can be subjected to erosion during the rainfall season from May to October in Taiwan. The soil sample for this study was collected from the surface (0–25 cm) of a mudstone site in the Tianliao area (E 120°22′58′′, N 22°53′02′′), Kaohsiung, Taiwan (Figure 1(b)). These mudstone soils could be classified as Typic Eutrustept according to the U.S. Soil Taxonomy [33].

The biochar used in this study was produced from rice hulls and supplied by the Industrial Technology Research Institute (ITRI) of Taiwan. Before charring, the rice hull was dried at 60°C for 24 h to <10% moisture and cut to a particle size of 2 cm. For pyrolysis, the samples were placed in a tubular furnace (ITRI, Tainan, Taiwan) equipped with a corundum tube (32 mm in diameter and 700 mm in length) with a N_2_ purge (1 L/min flow rate) to ensure an oxygen-free atmosphere. Heat treatments were performed in the temperature of 400°C. The heating rate was 5°C min^−1^. Temperature was maintained for 2 hrs before cooling to ambient temperature under N_2_ flow. The produced rice hull biochar was denoted as RHB-400 later in this study. After pyrolysis, the biochar was ground to enable it to pass through a 2 mm sieve, ensuring that all the biochar used in the experiments exhibited similar particle sizes. Rice hulls are considered agricultural waste, and they generate at least one million tons of waste per year in Taiwan. Recycling this waste and using it as an amendment for soil is favorable.

### 2.2. Analyses of Soil and Biochars

The soil samples were air-dried and ground to pass through a 2 mm sieve for subsequent analysis. The particle size distribution was determined using the pipette method [34]. The soil pH was determined using a 1 : 2.5 ratio of soil to water [35]. The content of carbon and nitrogen in the soil was measured using a Fisons NA1500 elemental analyzer (Thermo Electron Corporation, Wattham, Massachusetts, USA). The soil organic carbon content was determined using the wet oxidation method [36]. The cation exchange capacity (CEC) and exchangeable bases were measured using the ammonium acetate (pH = 7) method [37]. The calcium carbonate contents were determined by simple titrimetric method [38], where finely ground soil and biochar samples (2.0 g) were reacted with 2 M HCl for 16 h. The emitted CO_2_ in the reacted bottle was captured by NaOH and then was titrated by 0.1 M HCl, where titrated HCl volume was used to calculate CaCO_3_ contents. The bulk density (Bd) was determined using the core method [39], in which a stainless steel core (5 cm in diameter and 10 cm in height) was used to collect soil core to determine soil Bd in each sampling time during incubation. The saturated hydraulic conductivity (*K*_sat⁡_) was determined by applying the constant head method [40]. The *K*_sat⁡_ was measured in saturated soil packed in a core (the same core as with the determination of Bd). Water was added to the extended soil cores and a constant head was maintained until the effluence flow was reasonably steady. Then water depths inside and outside the cores were recorded; meanwhile, the effluent water volume was also recorded in a given time, and then *K*_sat⁡_ was calculated based on Darcy's equation. Soil penetration resistance (PR) was measured by using a portable hardness tester (Soil Hardness Meter, Yamanaka type, Daiki Rika Kogyo Co., Japan). Modified fast wetting in water, as proposed by [41], was used to measure the aggregate stability of 2 mm air-dried aggregates (35 g). A 4 cm amplitude was applied to a nest of sieves (>2000 mm, 1000–2000 mm, 500–1000 mm, 250–500 mm, 250–106 mm, and <106 mm) immersed in a container of tap water (101 mS/cm) during the 5 min of vertical movement. The material that remained in each sieve after wet shaking was carefully removed, and the mean weight diameter (MWD) of the aggregate size was calculated as follows:

(1) $$\begin{array}{c} \text{MWD}=\overset{n}{\underset{i=1}{\sum }}x_{i}w_{i}, \end{array}$$

where *n* is the number of sieves, *x* is the diameter, and *w* is the weight.

The SSAs of the soil and biochar were determined based on N adsorption isotherms at 77.3 K by using the Brunauer-Emmett-Teller (BET) equation [42] (PMI Automated BET Sorptometer BET-202A). The microbial biomass carbon (MBC) content in the soil was determined using fumigation and extraction [43, 44]. Fourier-transform infrared (FTIR) analysis was performed to test the quality of the biochar. Ground biochar (0.3–0.5 mg) was embedded in potassium bromide (KBr) pellets (99.5–99.7 mg) and measured using an FTIR spectrometer (VECTOR 22, Bruker, USA) with a 4 cm^−1^ resolution and 100 scans between the 4000- and 400-cm^−1^ wavenumbers [45]. To analyze C forms based on the FTIR spectra, we subtracted the background of the KBr window, automatically corrected the baseline and smoothed the spectra, identified the peaks, and normalized the spectra on a reduced portion of the wavenumbers (4000–500 cm^−1^).

The MBC was estimated only at the end of the incubation. Subsamples collected from the incubated treatments were fumigated with ethanol-free chloroform for 24 h at 25°C. After chloroform removal, the subsample was extracted using a 200 mL 0.5 M K_2_SO_4_ solution for 30 min. The organic carbon in the extract was measured by performing wet digestion using dichromate and titration using FeSO_4_.

Soil water retention curve was determined by a pressure plate method, in which a Buchner funnel with a ceramic plate connected with a burette by plastic tubing was used for this experiment. The measured soil water retention data at different matric potentials (500 cm, 1,000 cm, 3,000 cm, 6,000 cm, and 12,000 cm) and the air-dry water content with an assumed suction of 15,000 cm were used to fit the soil water retention model [46]:

(2) $$\begin{array}{c} \theta =\theta_{r}+\frac{\theta_{s}-\theta_{r}}{{\left[1+{\left(\alpha \left|h\right|\right)}^{n}\right]}^{1-1/n}}. \end{array}$$

Here *θ* is volumetric soil water content (cubic centimeters per cubic centimeter), *h* is the suction (centimeters), *θ*_*r*_ and *θ*_*s*_ are the residual water content and saturated water content (cubic centimeters per cubic centimeter), respectively, and *α* (per centimeter) and *n* are the fitting parameters. The plant available water content was calculated by the difference between the field capacity (the water content at suction of 330 cm) and the wilting point (the water content at suction of 15,000 cm). According to the definition of macropores [47], which are the pores with diameters >75 *μ*m (corresponding to *h* > −40 cm), the amount of macropores (cubic centimeters per cubic centimeter) was derived from the soil water retention curve.

### 2.3. Incubation Experiment

Incubation experiments were conducted to evaluate the effects of biochar on soil properties. The studied soil (15 kg) was placed in plastic pots (30 cm in width and 40 cm in length) and then well mixed with the biochar at application rates of 2.5%, 5%, and 10% (w/w). The mixed soils were wetted to the field water capacity (60% water holding capacity) including the control soil. The incubated pots were placed in a room at 28°C and weighed every 5 d to maintain the field water capacity. All treatments were conducted in triplicate. The total incubation time was 168 d, and the soils were analyzed at 3-week intervals to determine their chemical and physical properties.

### 2.4. Micromorphological Observations

After incubation, Kubiena boxes were employed in collecting undisturbed blocks of unamended and amended soil to create thin sections used for observing micromorphological features. After air-drying, vertically oriented thin sections measuring 2.5 cm × 5 cm × 30 *μ*m were prepared by Spectrum Petrographics (Vancouver, WA, USA). The thin sections were used to observe soil structures under a polarized microscope (Leica DM EP, Wetzlar, Germany). The microscale structure of the biochar sample was identified using optical microscopy with reflected light and, subsequently, scanning electron microscopy (SEM) (Hitachi, S-3000N, Japan). A backscattered electron image representing the mean atomic abundance in a black-and-white image was observed from the surface of the samples coated with Au. The mineral phases of the sample were identified using SEM and energy-dispersive spectroscopy (EDS) (Horiba, EMAX-ENERGY EX-200, Japan), in which an acceleration voltage and beam current of 15 kV and 180 pA, respectively, were applied in a 25 Pa vacuum with Au coating. The analyzed points were selected using backscattered electron images to avoid damaging the samples.

### 2.5. Soil Erosion Experiment

All treatments (the same treatments as with the incubation for analyses of soil physiochemical properties) were filled in a stainless steel cylinder (20 cm in diameter and 10 cm in height) and well put in the end of the erosional channel (20 cm in width and 90 cm in length). This set of the treated soils was also incubated for 168 d and then used for the erosion experiment by using rainfall simulator, which was with a height of 9.5 m, average diameter of 2.5 mm for rain drops, and an average terminal velocity of 8.5 m s^−1^ for rain drops. All devices and processes were according to the American Standard Testing Materials D7101-08 standard method [48]. The erosion experiment simulated a rainfall intensity of 80 mm h^−1^ and a slope gradient of 20°, because the 80 mm h^−1^ was the average rainfall intensity during typhoon season in the study area, and the slope gradient of 20° is the average slope gradient in this mudstone area.

### 2.6. Statistical Analysis

The rainfall experiments for all treatments were conducted in triplicate. The triplicate data were subjected to mean separation analysis by using a one-way ANOVA test with a significance level of *P* = 0.05. The differences between mean values were identified using Duncan's test. Pearson correlation coefficients were calculated to determine how the soil properties were related.

## 3. Results

### 3.1. General Properties of the Soil and Biochar

Table 1 lists some physicochemical properties of the soil and the biochars. The soil was silt clay loam and was characterized by very high pH and relatively low soil organic carbon (SOC) contents. Most of the total carbon was composed of calcite carbonate in the studied soil. Total nitrogen contents were also less than 0.10% in this soil. The CEC was around 10 cmole(+) kg^−1^, which corresponded to low amounts of SOC and clay fraction in the soil. Relatively high amounts of exchangeable Ca and Na were found in the soil, particularly in Ca (Table 1). Exchangeable Mg and K were less than 0.70 cmole(+) kg^−1^ in the soil.

**Figure 2:**
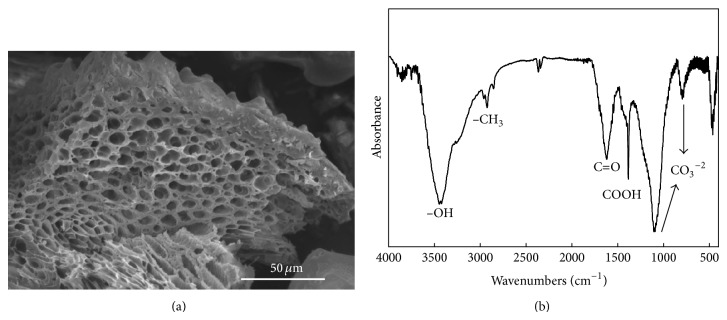
Scanning electron microscope (SEM) images (a) and Fourier-transform infrared (IR) spectrums of the rice hull biochar (RHB-400) (b).

The pH of the RHB-400 was 7.9. The contents of SOC, total C, and total N in RHB-400 were 3.27%, 33.0%, and 0.41%, respectively (Table 1). About 0.3% of CaCO_3_ was found in the RHB-400. The CEC of the RHB-400 (26.0 cmol kg^−1^) was higher than the soil. Table 1 further shows that exchangeable Mg and K were much higher in the biochars than in the studied soil. Specific surface area (SSA) was about 15.1 m^2 ^g^−1^. Regarding functional groups in the biochar, the FTIR analysis of biochar revealed large proportions of OH-stretching vibrations from the H-bonded hydroxyl (O–H) group (3365 cm^−1^) of phenol (aromatic compound) and carbonate (Figure 2).

### 3.2. Changes in Physical Properties after Biochar Application

Table 2 shows the physical properties of the biochar-amended soils after 168 d of incubation. The Bd of the amended soils significantly decreased from 1.59 Mg m^−3^ of the control to 1.40 Mg m^−3^, 1.35 Mg m^−3^, and 1.19 Mg m^−3^ of the treatments with 2.5%, 5%, and 10% of RHB-400, respectively. Besides, PR was also reduced from 14.5 kg cm^−2^ in the control sample to 1.2–6.3 kg cm^−2^ in the biochar-amended soils after 168 d incubation. In addition to obvious decreases in the Bd and PR, significantly higher total porosity was also found in the biochar-amended soils (44.5%–46.7%) compared to the control (38.3%), as well as the MWD of the soil aggregates, which increased from 1.15 mm to 1.74 mm–2.09 mm after biochar application.

### 3.3. Changes of Hydraulic Properties and Soil Loss Contents after Biochar Application

**Figure 3:**
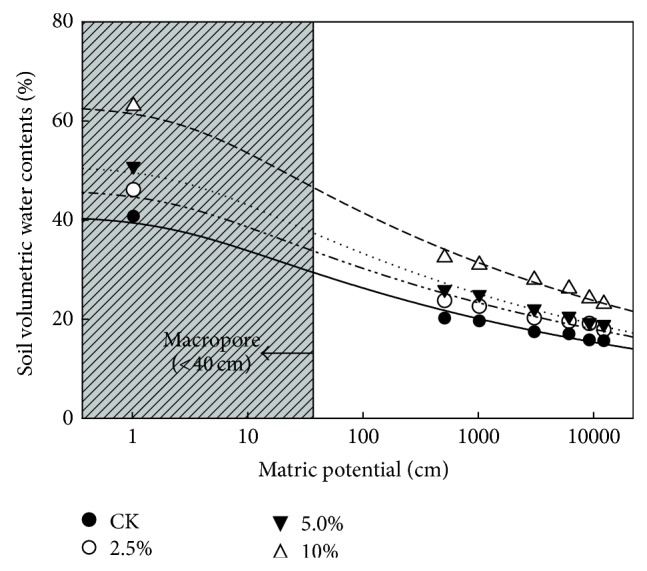
Water retention curves for the mudstones amended with rice hull biochars (RHB-400) after 168 d of incubation.

The biochars incorporation significantly increased water holding capacity (WHC) after 168 d incubation, particularly in the volumetric water contents (*θ*_*v*_) at low matric potentials (Figure 3). After incubation, the *θ*_*v*_ increased by 16% to 62% at −0.3 bar (field water capacity (FWC)) and by 16% to 52% at −15 bar (wilting point (WP)) in the biochar treatments (Table 2). Meanwhile, the available water content (AWC) also increased considerably by 18% to 89% in the biochar treatments. The highest contents of FWC, WP, and AWC were always found at 10% biochar-amended soils. As shown in Table 3 and Figure 3, macropore proportion (%) significantly (*P* < 0.05) increased after incorporation of biochars from 6.14% in the control to 6.44%–7.79% in the amended soils, particularly in 10% biochar treatment. Table 3 also shows that the *K*_sat⁡_ was determined only in the 10% biochar-amended soils after incubation. This indicated that a high biochar application rate (≥10%) improved the water conductivity of the soil. Soil loss contents at a simulated rainfall intensity of 80 mm h^−1^ and at a slope gradient of 20° are also presented in Table 3. The highest soil loss contents occurred in the control, and the lowest soil loss contents occurred in the amended soils with 10% biochar. The soil loss contents significantly decreased with increasing biochar application rate.

## 4. Discussion

### 4.1. Improvement of the Physical Properties of the Studied Soils

In this study, biochar incorporation obviously improved soil physical properties, particularly in soil strength and soil structure. Soil strength was commonly reflected by Bd and PR. The Bd of biochar-amended soils significantly decreased by 12% to 25% compared with the control. This was especially true for the 10% biochar treatment (Table 2). For agronomic aspect, high Bd (~1.60 g cm^−3^) of the control might restrict root extensibility and therefore inhibit growth of crops [9]. The Bd in the biochar-amended soils (<1.4 g cm^−3^) has been accepted for tillage and crop growth (Table 2) [49]. Furthermore, 2.5% biochar incorporation was recommended in this study, because PR might be much suitable for plant growth in this treatment (2.0–6.0 kg cm^−2^) which was proposed by [49].

Soil structure was always determined by porosity and soil aggregates. In the present study, porosity and aggregate size were increased after the biochar application (Table 2). The obvious change of porosity in this study was considered as the formation of macropores and rearrangement of soil particles. Based on the study, macropores (>75 *μ*m in diameter) increased obviously after biochar incorporation (Table 3), and this might be attributed to the dilution effect [12] and soil particle rearrangement [10]. As shown in Table 3 and Figure 3, macropores obviously increased by 4% to 27% in the biochar-amended soils after incubation. This finding was similar to the result of Lei and Zhang [50] who indicated 5%–35% increases of macropore proportion in sandy loam soil after application of 5% wood biochar. They also proposed that change of porosity in biochar-amended soils resulted from the rearrangement of soil particles. Meanwhile, micropores were also obviously increased after incorporation of biochar (Figure 3, Table 3). Numerous micropores in the biochar itself observed in Figure 2(a) might be responsible for a large increase of micropores in the biochar-amended soils [12, 13].

**Figure 4:**
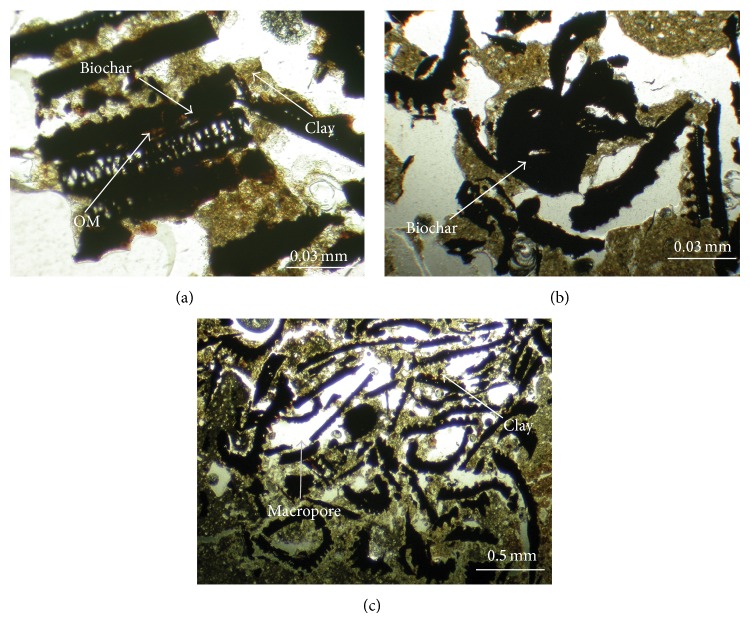
Micrographs of thin sections of soils amended with biochar: (a) soil aggregates with 5% application rate of RHB-400 with plain polarized light (PPL); (b) soil aggregates with 5% application rate of RHB-700 with plain polarized light (PPL); (c) soil aggregates with 10% application rate of RHB-700 with plain polarized light (PPL).

Except for obvious increases of macro- and micropores in the amended soils, rearrangement of soil particles also obviously coarsens soil aggregates in this study, particularly in the 10% biochar treatment (Table 3). Regarding increases of MWD of soil aggregate after biochar application in this study, the increases in the TOC contents and microbial activity might be the critical reasons, which also agreed with [10, 23]. The present study revealed that the TOC content increased from 0.7% to 1.5–3.7% in the biochar-amended soils, and the MBC content also increased, especially with the 10% biochar treatments (Table 4). The formation of soil aggregates might result from the interaction between the surface of soil particles and functional groups (e.g., phenolic and carboxylic groups (Figure 2)) contained in the biochar [12]. Observations of the microstructures in the biochar-amended soils indicated that soil particles (clay) seemed to be adsorbed on the biochar, forming microaggregates (Figures 4(a) and 4(b)) and then continuing to combine with other soil-biochar complexes to form new macroaggregates (Figure 4(c)). Jien and Wang [10] also observed a similar process when they incorporated a wood biochar into the highly weathered soil.

### 4.2. Improvement of Hydraulic Characteristics by Biochar Application

Poor WHC implicated insufficiently available water in soils for plant growing. In this study, the poor WHC of the mudstone soil was attributed to less organic substance contents and poor soil structure. Not only increase of organic substances, but also improvement of soil structure was found after biochar incorporation into the soil. The FWC, WP, and AWC also significantly increased by 60%, 44%, and 104%, respectively, in the amended soils as compared with the control after incubation (Table 3, Figure 3). This further implicated that plants could obtain much water in this mudstone area where there is always water deficiency during growing season. Lei and Zhang [50] indicated that biochar application directly increased the WHC through the inner surface area of the biochar and indirectly increased the WHC by facilitating the formation of soil aggregates and macropores.

In this study, Figure 3 shows that *θ*_*v*_ in the biochar-amended soils was higher than in the control at any matric potential throughout the incubation period. This further implicated that macropores and micropores obviously increased after biochar application. The micromorphological observations in our study also indicated the rearrangement of soil particles in the biochar-amended soils (Figure 4). The increase of WHC at higher matric potentials can be ascribed to the extremely high inner SSAs of the biochar and the textural pores produced by the formation of aggregates (complexes of the biochar and soil particles) (Figures 4(a) and 4(b)). Rawls et al. [51] indicated that soil water retention was strongly influenced by organic matter at −0.3 to −15 bar. Liu et al. [52] stated that the WHC was determined by organic matter content and intraaggregate pores at matric potentials of 0.1 and 5 bar. Our results corresponded with the findings of Liu et al. [52], who determined that larger aggregates retained more water than smaller aggregates at matric potentials of 0.1 and 5 bar because of intraaggregate pores. Kutílek et al. [53] indicated that water retention at lower matric potential depends on the contents of larger pores, which is strongly influenced by soil structure, while water retention at higher matric potentials is affected more by soil texture and surface area. The formation of a larger inner surface area and the more porous structure of the RHB produced by a higher temperature could be the main reason why the RHB-700 treatments retained more water than the RHB-400 treatments did; this explanation was consistent with those of Verheijen et al. [54] and Lei and Zhang [50].

In addition, the *K*_sat⁡_ increased in the 10% biochar-amended soil. The *K*_sat⁡_ results followed a trend similar to that of the macropore results in the biochar-amended treatments. The large increase in *K*_sat⁡_ in the 10% biochar-amended soils may be attributed to the increase in macropores (>−0.04 bar), which was observed at low matric potentials (Figure 3). Macropores might be formed through dilution effects and the rearrangement of soil particles when the surfaces of clay and biochar interact [10]; this process was evidenced in our micromorphological observations (Figure 4). Herath et al. [12] proved that the *K*_sat⁡_ was determined by the macropore proportions, particularly in poorly drained soils. Lei and Zhang [50] also indicated that *K*_sat⁡_ increased after woodchip and dairy manure biochar was added because of the improvements in soil aggregation and macropores.

### 4.3. Improvement of Soil Erosion Potential by Biochar Application in Mudstone Soil

From the result of correlation analysis, significantly positive correlations were presented between Bd and soil loss contents (*r* = 0.91, *P* < 0.01) and between PR and soil loss contents (*r* = 0.93, *P* < 0.01) (Table 5). Reduction of soil strength might be the major factor in decreasing of the soil loss contents in the biochar-amended soil. Soil crusting can increase Bd and PR and therefore increased the runoff and sediment yields [55]. Singer and Shainberg [56] also explained that the formation of crusting and sealing increases the erosion rate and sediment yield on slopelands. In this study, the dilution effect and the formation of microaggregates or macroaggregates induced by biochar incorporation might be the key factors to reduce crusting. Reduction of soil crusting therefore increased infiltration ability, followed by the decreasing of soil loss contents (Table 3).

Except for reduction of soil strength in the amended soils, the change of soil structure including increase of porosity and aggregate stability could be identified as a minor factor to reduce soil loss contents in the studied soil. Soil aggregation stability is generally considered to be related to the erosion of degraded soils [31, 52]. An et al. [57] indicated that the content of stable aggregates most strongly reflected the ability of soil to resist erosion on the Loess Plateau. In this study, porosity (*r* = −0.72, *P* < 0.01), soil aggregate size (*r* = −0.80, *P* < 0.01), macropores (*r* = −0.85, *P* < 0.01), and micropores (*r* = −0.82, *P* < 0.01) were significantly negatively correlated to soil loss contents (Table 5). Redistribution of soil particles by applying biochar increased the soil aggregate stability to prevent the soil particles from detaching and increased the number of macropores, facilitating the permeability of the soil. Liu et al. [52] indicated that applying biochar may reduce soil erosion by improving the aggregate stability of soil; these results corresponded with our results. The unamended mudstone soils were highly susceptible to water erosion because of their unstable aggregates. Approximately 80% of the soil aggregates in the unamended mudstone soils examined in this study exhibited diameters that were less than 0.05 mm, indicating that the aggregates in the hardsetting mudstone soils were unstable. This result corresponded with the description by Le Bissonnais [41], who indicated that soils were considered highly unstable when their MWDs were < 0.4 mm. Cantón et al. [58] stated that aggregate stability and macroaggregate formation were crucial for maintaining soil porosity and improving soil erosion. Barthès and Roose [59] reported that soil losses were negatively correlated with stable macroaggregate (>0.2 mm) contents (*r* = 0.99, *P* < 0.01) in topsoils at a certain simulated rainfall intensity (60 mm h^−1^).

## 5. Conclusion

Agronomy and engineering solutions, including tillage, forest restoration, soil erosion prevention, tunneling, and road construction in mudstone areas, are worldwide problems, because of the poor physical properties of these soils. Our results indicated that applying rice hull biochar can effectively improve poor soil physiochemical properties and reduce the soil erosion potential in mudstone areas. Our results further indicated that biochar incorporation could significantly reduce soil strength by decreasing the Bd and the PR after a short-term incubation. Moreover, soil structure could also be changed by increasing porosity, aggregate size, and FWC from 6% to 18%, 0.3 to 1.05 mm, and 3.8% to 17%, respectively. Meanwhile, biochar application could be expected to significantly reduce soil loss contents by 35% to 90% under an extreme rainfall event based on the results.

## Figures and Tables

**Table 1:** General properties of the studied soil and the biochar.

Properties	Soil	Rice hull biochar
Sand (%)	7.90 ± 1.20^a^	ND
Silt (%)	58.6 ± 5.30	ND
Clay (%)	33.5 ± 2.70	ND
pH	8.70 ± 0.05	7.90 ± 0.02
SOC (%)	0.49 ± 0.05	3.27 ± 0.05
Total carbon (%)	3.35 ± 0.53	33.0 ± 0.61
Total nitrogen (%)	0.06 ± 0.02	0.41 ± 0.03
CaCO_3_ (g/kg)	22.1 ± 1.29	4.56 ± 0.53
CEC (cmol kg^−1^)	9.83 ± 0.27	26.0 ± 1.13
Ca (cmol kg^−1^)	26.2 ± 1.18	2.44 ± 0.18
Na (cmol kg^−1^)	1.58 ± 0.07	1.26 ± 0.35
Mg (cmol kg^−1^)	0.63 ± 0.03	1.91 ± 0.44
K (cmol kg^−1^)	0.37 ± 0.03	18.7 ± 0.30
SSA (m^2^ g^−1^)	ND	15.1

ND: not determined; SOC: soil organic carbon estimated by wet oxidation method; CEC: cation exchange capacity; Ca, Mg, K, and Na were determined as the exchangeable forms; SSA: specific surface area.

^a^standard deviation.

**Table 2:** Physical properties of the studied soil with different biochar treatments after 168 days.

Biochar	AR	Bd	PR	Porosity	MWD
		Mg m^−3^	kg cm^−2^	%	mm
Control	0%	1.59 ± 0.03^e^	14.5 ± 1.47^i^	38.3 ± 2.44^a^	1.15 ± 0.30^ab^
RHB-400	2.5%	1.40 ± 0.03^a^	6.30 ± 0.19^h^	44.5 ± 1.97^ab^	1.74 ± 0.28^cde^
	5.0%	1.35 ± 0.01^a^	2.20 ± 0.16^ef^	46.7 ± 0.54^bc^	1.77 ± 0.35^cde^
	10%	1.19 ± 0.06^d^	1.20 ± 0.13^bc^	45.3 ± 2.55^bc^	2.09 ± 0.17^de^

Values followed by the same letter within a column are not significantly different at *P* < 0.05 level based on Duncan's test.

AR: application rate; Bd: bulk density; PR: penetration resistance; MWD: mean weight diameter of soil aggregates.

**Table 3:** Mean soil volumetric moisture contents measured at different matric potentials, macropores, and saturated water conductivity of the control and biochar-amended soils after incubation (*n* = 3).

Biochar	AR	Macropore	Micropore	FWC	WP	AWC	*K*_sat⁡_	SLC
				%			cm day^−1^	
Control	0%	6.14 ± 0.14^a^	29.3 ± 0.42^a^	22.7 ± 1.17^a^	16.3 ± 0.68^a^	6.41 ± 0.25^a^	NA	580 ± 110^a^
RHB-400	2.5%	6.44 ± 0.12^b^	32.8 ± 0.28^b^	26.5 ± 1.08^b^	18.9 ± 0.52^b^	7.54 ± 0.50^b^	NA	378 ± 36.8^b^
	5.0%	6.38 ± 0.22^b^	35.4 ± 2.71^c^	29.6 ± 1.45^bc^	19.8 ± 0.88^b^	9.74 ± 1.78^b^	NA	120 ± 52.6^c^
	10%	7.79 ± 1.15^c^	45.4 ± 2.99^d^	36.9 ± 2.60^d^	24.8 ± 0.42^c^	12.1 ± 0.18^c^	2.17 ± 2.03	60.2 ± 43.0^c^

AR: application rate; FWC: field water content; WP: wilting point; AWC: available water content; *K*_sat⁡_: saturated hydraulic conductivity; SLC: soil loss contents.

Values followed by the same letter within a column are not significantly different at *P* < 0.05 level based on Duncan's test. NA: not available.

**Table 4:** Soil organic carbon contents and microbial biomass carbon of the studied soils after 168 d incubation.

Treatments	Application rate	Organic carbon contents^∗^	Microbial biomass carbon
		g kg^−1^	mg kg^−1^
Control	0%	0.68 ± 0.05^a^	31.3 ± 1.18^a^
RHB-400	2.5%	1.51 ± 0.08^b^	33.7 ± 2.08^a^
	5.0%	2.08 ± 0.03^c^	53.8 ± 25.2^ab^
	10%	3.71 ± 0.10^d^	129 ± 26.3^c^

^*∗*^Organic carbon contents were determined by differences between contents of total carbon and carbon derived from CaCO_3_. Different letters beside the values mean significant difference (*P* < 0.05) at the same column.

**Table 5:** Pearson's correlation coefficients among soil properties at the end of incubation time (after 168 d), *n* = 12.

	Bd	PR	Porosity	MWD	MaP	MiP	AWC	SLC
Bd	1.00							
PR	0.90^∗∗^	1.00						
Porosity	−0.73^∗∗^	−0.69^∗^	1.00					
MWD	−0.87^∗∗^	−0.65^∗^	0.68^∗^	1.00				
MaP	−0.95^∗∗^	−0.88^∗∗^	0.79^∗∗^	0.80^∗∗^	1.00			
MiP	−0.94^∗∗^	−0.84^∗∗^	0.66^∗∗^	0.82^∗∗^	0.97^∗∗^	1.00		
AWC	−0.95^∗∗^	−0.84^∗∗^	0.72^∗∗^	0.83^∗∗^	0.97^∗∗^	0.95^∗∗^	1.00	
SLC	0.91^∗∗^	0.93^∗∗^	−0.72^∗∗^	−0.80^∗∗^	−0.85^∗∗^	−0.82^∗∗^	−0.88^∗∗^	1.00

^∗^ *P* < 0.05; ^∗∗^ *P* < 0.01.

Bd: bulk density; PR: penetration resistance; MWD: mean weight diameter of soil aggregates; MaP: macropores; MiP: micropores; AWC: available water content; SLC: soil loss contents.
